# Vascular Dysfunction following Polymicrobial Sepsis: Role of Pattern Recognition Receptors

**DOI:** 10.1371/journal.pone.0044531

**Published:** 2012-09-07

**Authors:** Stefan Felix Ehrentraut, Anne Dörr, Heidi Ehrentraut, Ralph Lohner, Sun-Hee Lee, Andreas Hoeft, Georg Baumgarten, Pascal Knuefermann, Olaf Boehm, Rainer Meyer

**Affiliations:** 1 Department of Anaesthesiology and Intensive Care Medicine, University Hospital Bonn, Bonn, Germany; 2 Institute of Physiology II, University of Bonn, Bonn, Germany; University of São Paulo, Brazil

## Abstract

**Aims:**

Aim was to elucidate the specific role of pattern recognition receptors in vascular dysfunction during polymicrobial sepsis (colon ascendens stent peritonitis, CASP).

**Methods and Results:**

Vascular contractility of C57BL/6 (wildtype) mice and mice deficient for Toll-like receptor 2/4/9 (TLR2-D, TLR4-D, TLR9-D) or CD14 (CD14-D) was measured 18 h following CASP. mRNA expression of pro- (Tumor Necrosis Factor-α (TNFα), Interleukin (IL)-1β, IL-6) and anti-inflammatory cytokines (IL-10) and of vascular inducible NO-Synthase (iNOS) was determined using RT-qPCR. Wildtype mice exhibited a significant loss of vascular contractility after CASP. This was aggravated in TLR2-D mice, blunted in TLR4-D animals and abolished in TLR9-D and CD14-D animals. TNF-α expression was significantly up-regulated after CASP in wildtype and TLR2-D animals, but not in mice deficient for TLR4, -9 or CD14. iNOS was significantly up-regulated in TLR2-D animals only. TLR2-D animals showed significantly higher levels of TLR4, -9 and CD14. Application of H154-ODN, a TLR9 antagonist, attenuated CASP-induced cytokine release and vascular dysfunction in wildtype mice.

**Conclusions:**

Within our model, CD14 and TLR9 play a decisive role for the development of vascular dysfunction and thus can be effectively antagonized using H154-ODN. TLR2-D animals are more prone to polymicrobial sepsis, presumably due to up-regulation of TLR4, 9 and CD14.

## Introduction

The innate immune system detects and binds pathogen associated molecular patterns (PAMPs) [1] by pattern recognition receptors (PRRs), including the Toll-like receptors (TLRs) [2]. The consecutive transcellular signaling initiates an inflammatory response and can culminate into the clinical picture of sepsis [3], [4]. The high mortality of this disease is mostly due to an evolving multi organ dysfunction caused in part by malperfusion due to reduction of total peripheral resistance [5]. Different sepsis models have been established to broaden the understanding of the underlying molecular mechanisms. These approaches include stimulation with distinct TLR agonists, like the TLR4 ligand lipopolysaccharide (LPS) from Gram-negative bacteria [6] or the TLR2 agonist lipoteichoic acid (LTA) from Gram-positive bacteria [7]. It has been shown that LPS induces vascular dysfunction in mice; this can be attenuated using the TLR4 antagonist Eritoran *in vitro* and *in vivo* enhancing vascular function and cardiac output [8], [9]. Vascular dysfunction is further aggravated by LPS-dependent impairment of cardiac contractility culminating in septic heart failure [10]. Furthermore, it has been shown, that the soluble pattern recognition receptor for LPS CD14 is necessary for recognizing low amounts of LPS [11] and CD14-deficient mice are nearly insensitive to Gram-negative septic shock [5]. However, clinical sepsis is often not caused by a mono-virulent infection but by a polymicrobial stimulus. This type of sepsis is much more complex although it appears to be mediated through TLRs as well [12]. A multibacterial setting of sepsis is likely to stimulate several TLRs simultaneously, due to the appearance of various cell wall components and bacterial DNA. Interestingly, different experimental approaches to induce sepsis appear to stimulate variable pathways for the induction of vascular dysfunction [13], [14].

**Figure 1:**
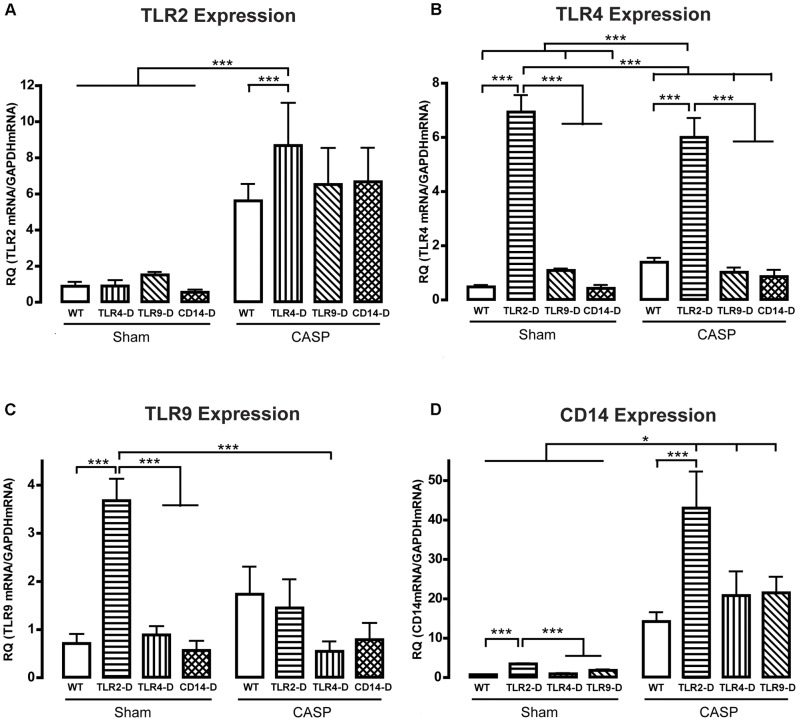
Effects of CASP and expression of PRR expression in the murine vessel wall. PRR mRNA expression during control conditions and following CASP-surgery (TLR2 (A), TLR4 (B), TLR9 (C), CD14 (D)). TLR2-D mice showed significantly higher baseline levels of TLR4 (B), TLR9 (C) and CD14 (D) compared to the other groups. All PRRs appeared to be regulated after CASP; however, only TLR2 and CD14 mRNA expression reached the level of significance (A, D; *p<0.05; **p<0.01; ***p<0.001; n≥5 animals in each group; mean ± SEM).

Contrary to experimental endotoxaemia, where loss of vascular function is associated with iNOS-dependent pathways [8], [10], work by Wang and colleagues indicates that experimental polymicrobial sepsis, utilizing the caecal ligation and puncture (CLP) model, resulted in loss of vascular contractility independent of NO production [13]. Hence, there might be differences in the organism’s reaction to multibacterial stimuli in regard to which specific TLR is stimulated. There is evidence in the literature, that TLR9 is crucial for the induction of polymicrobial sepsis. Plitas et al. showed that TLR9-deficient mice have lower serum pro-inflammatory cytokine levels and a higher bacterial clearance during polymicrobial sepsis resulting in improved survival [15]. Furthermore, it has been shown that TLR9 antagonism attenuates sepsis-induced kidney failure [16].

The aim of this study was to further elucidate the role of different TLRs for their specific impact on vascular contractility during polymicrobial sepsis. Furthermore, we were interested, whether pharmacological inhibition of the relevant receptor might be a suitable target for treatment of vascular dysfunction during polymicrobial sepsis.

## Materials and Methods

### Animal Handling and Care

12- to 14-week-old C57BL/6 (wild-type, WT) mice of both genders were purchased from Charles River (Sulzfeld, Germany). TLR2- (TLR2-D), TLR4- (TLR4-D), CD14- (CD14-D) and TLR9-deficient (TLR9-D) mice were kindly provided by Professor Shizuo Akira (Osaka University, Osaka, Japan) and backcrossed to a C57BL/6 background. Mice were housed in individually ventilated cages with free access to rodent chow and water with 12 hours automated light/dark cycles.

**Figure 2:**
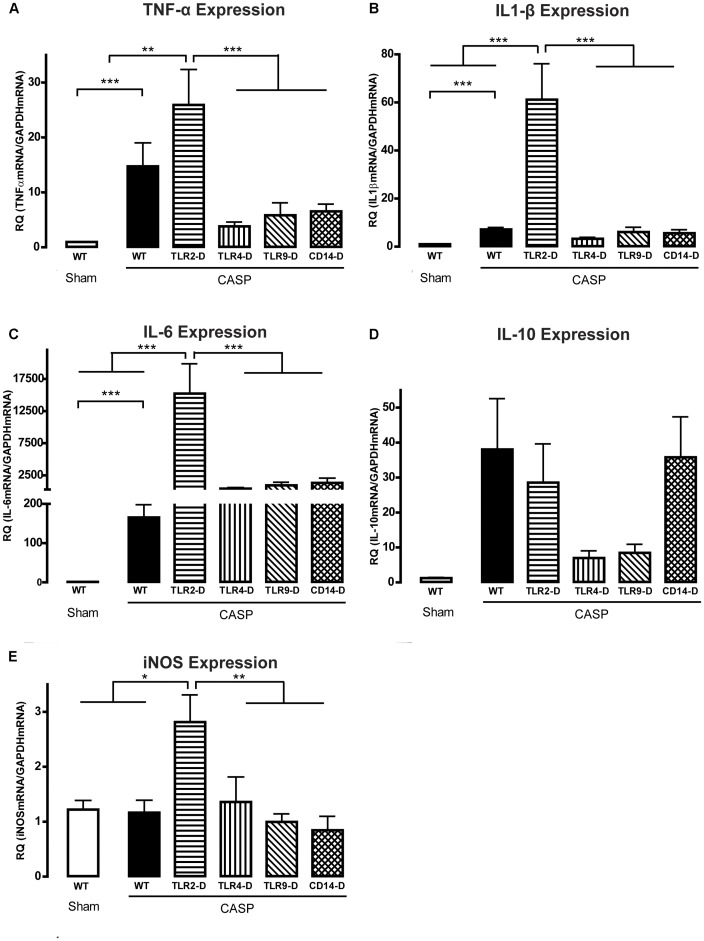
CASP induced aortic cytokine and iNOS production. **A** Pro-inflammatory TNF-α was significantly up-regulated in WT mice compared to sham animals. CASP-surgery in TLR2-D animals lead to even higher levels of TNF-α compared to all other groups. **B, C** IL-1β and IL-6 were significantly up-regulated in TLR2-D animals after CASP compared to the other strains. **D** CASP induced a non-significant up-regulation of the anti-inflammatory cytokine IL-10 in WT, TLR2-D and CD14-D mice. **E** CASP induced a significant up-regulation of iNOS in TLR2-D mice only. (*p<0.05; **p<0.01; ***p<0.001; n≥5 animals in each group; mean ± SEM).

### Ethics Statement

All experiments were performed in accordance with the Guide for the Care and Use of Laboratory Animals published by the US National Institutes of Health (NIH Publication No. 85–23, revised 1996). Treatment protocols were approved by the district government of Northrhine-Westphalia, here the “State office for protection of environment, nature and consumers” in Recklinghausen is the responsible agency. The approval number for this project is: LANUV 8.87-51.04.20.09.391.

### Colon Ascendens Stent Peritonitis (CASP)

The CASP-model was chosen to induce polymicrobial sepsis [17]. Mice were anaesthetized with 1.5vol% isoflurane (Forene, Abbot GmbH, Ludwigshafen, Germany) with an oxygen flow of 1 l/min using a nasal cone. Sufficient anesthesia was ascertained by non-responsiveness to a tail pinch. A small midline incision was performed to open the peritoneal cavity. The caecum was exposed under sterile conditions, followed by insertion of an 18 gauge stent into the colon ascendens, which was kept in place with 7/0 Ethilon thread (Ethicon, Norderstedt, Germany). After ascertaining free passage of feces through the stent, to ensure a save intraluminal placement, the caecum was relocated into the peritoneal cavity and the abdomen was closed with Ethilon 5/0 thread. Sham-operated animals underwent the same procedure (laparotomy and caecal exposure) without stent insertion. To prevent hypothermia during surgery, the animal was placed on a heating pad and, in addition, body temperature was controlled via a rectal probe regulating an infrared lamp.

**Figure 3:**
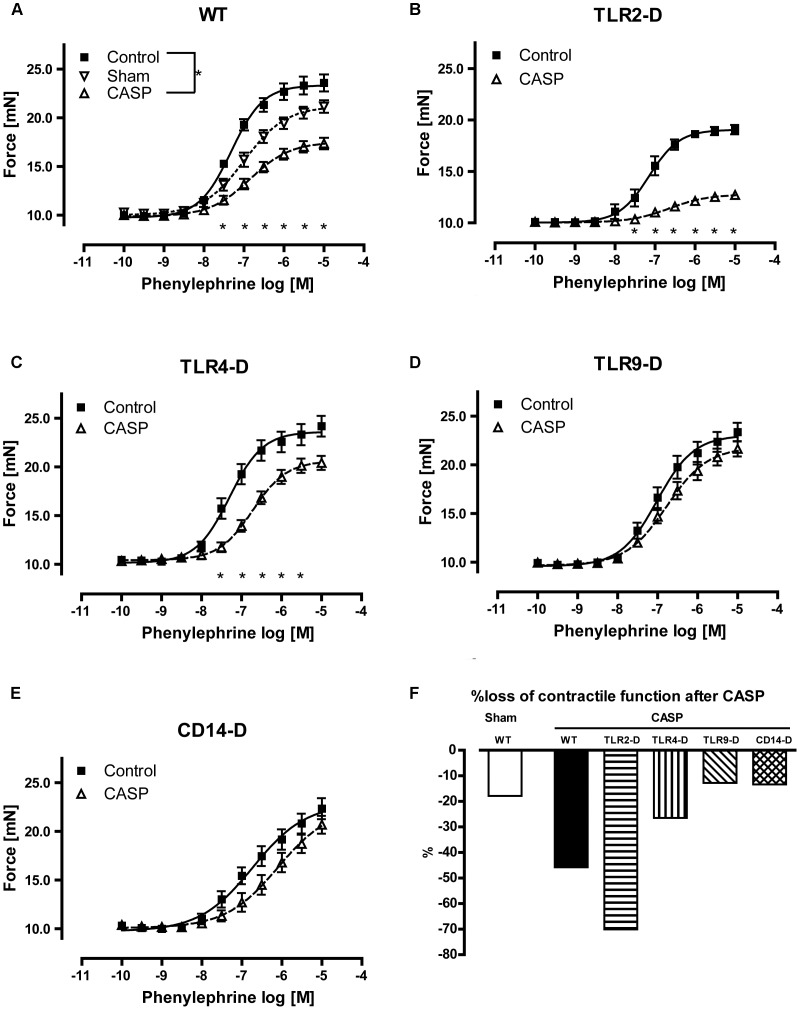
Arterial contractility 18 hours after CASP. **A** A significant hypocontractility of aortic rings in CASP- WT animals, but not in sham-operated WT animals was observed at phenylephrine concentrations of 10^−7,5^ to 10^−5^M. **B** TLR2-D mice exhibited a significantly lower maximal contractile response compared to WT animals (TLR2-D 19.07±0.454 mN vs. WT 23.59±0.872 mN; p<0.05). **C** Significant reduction of vascular contractility following CASP was also observed in TLR4-D mice compared to controls (p<0.05), but it was less severe than in WT animals. **D, E** CASP did not influence vascular contractility in TLR9-D and CD14-D mice, however CD14-D mice had a significantly higher EC50 of phenylephrine (Table S1). **F** Standardized maximal loss of vascular contractility. In WT mice sham surgery led to a 17% decrease of vascular contractility, compared to 45% after CASP-treatment. TLR2-D animals lost 70% of their maximum contractile response, whereas CASP-treated TLR4-D mice lost only 27% and CD14-D as well as TLR9-D mice were insensitive to CASP (*p<0.05; n≥5 animals in each group; mean ± SEM).

All mice received a single subcutaneous injection of 0.1 mg/kg bodyweight buprenorphine (in approx. 500 µl sterile PBS) for analgesia and fluid resuscitation.

### Bacterial Culture

At the beginning of experiments and 18 h after CASP the peritoneal cavity was flushed with 3 ml of a sterile phosphate buffer solution (PBS) and aspirated into a sterile syringe. Aliquots of a serial log dilution of this peritoneal lavage fluid were plated on Columbia blood agar and MacConkey agar to select for either Gram-positive or Gram-negative bacteria. The cell culture dishes were incubated for 48 h at 37°C and the amount of colony forming units (CFU) evaluated.

**Figure 4:**
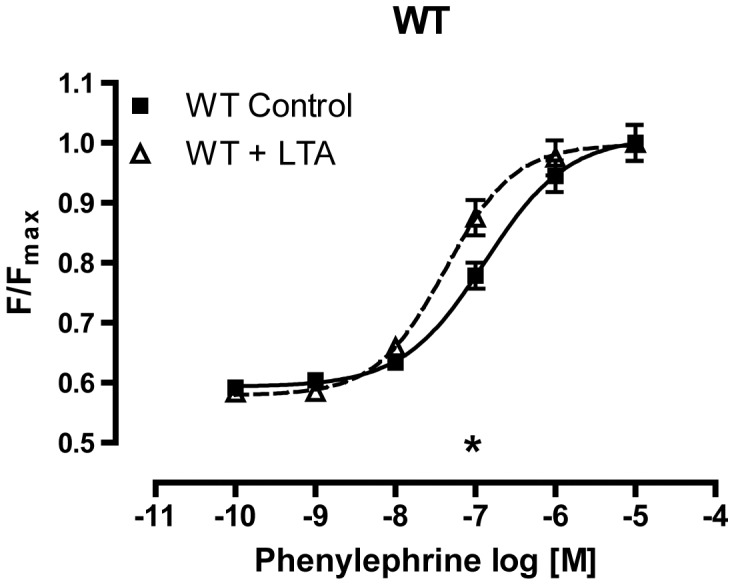
Influence of TLR2 agonism on aortic contractility. LTA application to WT mice increased contraction force at 10^−7^ M phenylephrine. Results were standardized to the maximum contraction force, which was not different between both groups (*p<0.05; n≥5 animals in each group; mean ± SEM).

### Injection of TLR9 Antagonist H154-thioate

The immunosuppressive oligodeoxynucleotide (ODN) H154-thioate (5′-CCTCAAGCTTGAGGGG-3′) acts specifically via TLR9 [18]. In our setting we injected 0.2 mg/kg BW (approx. 50 µg/mouse) into the tail vein 24 h prior to CASP surgery to mimic a prophylactic preoperative treatment option.

**Figure 5:**
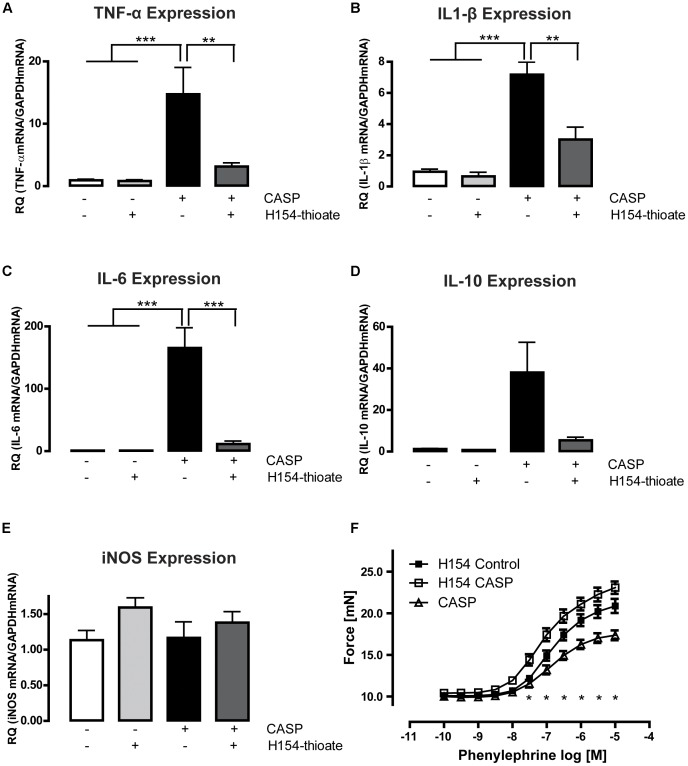
Prevention of CASP induced aortic cytokine and iNOS production following TLR9 antagonist treatment. **A-E** 24 h of exposure to the synthetic TLR9 antagonist H154-thioate did not induce mRNA expression of the investigated cytokines, but prevented the CASP-dependent rise in mRNA expression of inflammatory mediators. There was no observable influence of H154-thioate on iNOS expression. **F** H154-thioate treatment prior to CASP completely prevented the CASP-induced arterial hypocontractility observed in WT animals (F) (*p<0.05; **p<0.01; ***p<0.001; n≥5 animals in each group; mean ± SEM).

### LTA Treatment Protocol

Purified Staphylococcus aureus lipoteichoic acid (LTA; 15 mg/kg; InvivoGen, San Diego, USA) dissolved in sterile, endotoxin free LAL water (InvivoGen) was injected intraperitoneally (i.p.). Phosphate buffered saline injections were used as negative controls. 6 h after stimulation aortic ring contractility was measured as described below.

### Mulvany Myograph and Contractility Studies

18 h after CASP procedure, mice were put under deep isoflurane anaesthesia (>2vol%, sufficient depth was ascertained by non-response to tail pinch) and sacrificed by decapitation to prevent structural stress to the aorta. Vessels were then prepared and measured as described previously [8]. Isometric contractions were digitized using a Powerlab (ADInstruments GmbH, Spechbach, Germany) and continuously recorded using Chart for Windows (Version 5.5.5, ADInstruments).

### Cytokine mRNA Isolation and RT-qPCR

Tissue was harvested 18 h after CASP or sham surgery. The aortae were homogenized and RNA was isolated using the thiocyanate-phenol-chloroform method [19]. Afterwards, RNA was purified using the Micro-to-Midi Total RNA purification kit (Invitrogen, Carlsbad, CA, USA) and reversely transcribed using High capacity cDNA reverse transcription kit (Applied Biosystems, Darmstadt, Germany; Part No. 4368814) according to the manufacturer’s protocol. Relative RT-PCR for Actin, IL-1, IL-6, Il-10, TNFα, iNOS, TLR-2, TLR-4, TLR-9, and CD14 was performed using TaqMan Gene expression Master Mix (Applied Biosystems, Part No. 4369016) with the primers previously described [8], [20]. The reaction was processed in a TaqMan PCR system (Applied Biosystems), and the results were analyzed by calculating the ratio of target accumulation over GAPDH accumulation.

### Statistical Analysis

Numerical results are given as means ± standard error of the mean (SEM) of n observations, n being the number of tested animals. For analysis of numerical data, Student’s unpaired *t* test or 1-Way ANOVA with Newman-Keuls multiple comparison *post-hoc* test were used where appropriate using Prism 4.03 (GraphPad Software Inc., San Diego, CA, USA). Probability values <0.05 (*), <0.01 (**), <0.001 (***) are indicated.

## Results

### Clinical Symptoms and Mortality Following CASP

18 h after CASP surgery, clinical manifestations of infection were observed. These findings included lethargy, nasal and ocular discharge and piloerection, beginning as early as 2 h after surgery, and were most prominent in TLR2-D mice and not observable in TLR9-D or CD14-D animals. Mortality in TLR2-D mice increased to 30% after 18 h compared to zero percent in WT, TLR4-D, TLR9-D or CD14-D mice. Sham operation did not induce any obvious clinical symptoms.

### Intraperitoneal Bacteria Counts

To validate the polymicrobial sepsis model, bacterial cultures were prepared and evaluated regarding the amount of bacteria in peritoneal lavages.

We did not detect any bacteria in the peritoneal cavity of WT sham animals. CASP-surgery induced a significant increase in the amount of bacteria in the peritoneal cavity of CASP-WT leading to 3.044×10^7^ (±1.5539×10^7^, n = 10; p<0.05 vs. 0 h) Gram-negative and 1.224×10^6^ (±3.40552×10^5^, n = 5; p<0.05 vs. 0 h) Gram-positive CFUs after 18 h. This indicates liberation of faeces into the abdominal cavity after CASP surgery. Furthermore, there were no significant differences in the amount of bacteria released to the peritoneal cavity of TLR2-D or TLR9-D animals (TLR-9D: gram-positive 3.900×10^6^±1.444×10^6^, gram-negative 2.678×10^6^±1.048×10^6^ n = 5; TLR2-D: gram-positive 2.8×106±8.74×10^5^, gram-negative 2.702×10^7^±1.343×10^7^ n = 5).

### TLR4, -9 and CD14 were Up-regulated in Aortae of TLR2-D Animals Previous to CASP Surgery

PRR-dependent signal strength depends in part on the amount of receptor expression. Therefore, mRNA expression of PRRs in the aortic wall was investigated in a first step. TLR2-D mice showed a significantly higher baseline expression of TLR4, TLR9 and CD14 (Fig. 1B–D; the ablated receptor was not monitored in the respective group). In contrast, the baseline expression of PRRs in the aorta was not significantly influenced by gene ablation of TLR4, -9 and CD14 (Fig. 1A–D). CASP surgery led to a significant elevation of TLR2 mRNA in TLR4-D animals (Fig. 1A) and a significant, 2.5-fold up-regulation of CD14 mRNA (Fig. 1D) in all groups. The observed significantly higher baseline expression of TLR4 mRNA in TLR2-D animals compared with all other groups remained unaffected by CASP surgery (Fig. 1B). But the high baseline expression of TLR9 mRNA in aortae of TLR2-D animals was blunted by 18 h of polymicrobial stimulation, as significant differences to WT animals could not be detected any more (Fig. 1C). The co-receptor CD14 was already expressed to the highest level before CASP surgery in TLR2-D sham animals. During polymicrobial sepsis, the amount of CD14 mRNA was significantly up-regulated in all genotypes. Again, CD14 mRNA expression reached the highest level in TLR2-D animals, but was not significantly higher than in the other TLR-deficient groups after CASP surgery (Fig 1D).

### CASP-induced Cytokine Induction was Significantly Increased in TLR2-D Mice

To monitor vascular inflammation, we investigated the pro- and anti-inflammatory cytokine levels in the aortic wall. Baseline cytokine levels did not differ between genotypes (not shown). In WT mice CASP-surgery induced a significant increase in the mRNA-level of the pro-inflammatory cytokine TNF-α (Fig. 2A). None of the investigated pro-inflammatory cytokines was significantly up-regulated due to CASP in TLR4-D, TLR9-D or CD14-D mice (Fig. 2A–C).

In contrast, CASP caused a significantly higher expression of TNF-α, IL-1β and IL-6 mRNA in TLR2-D mice compared to all other groups (Fig. 2A–C). The anti-inflammatory cytokine IL-10 was up-regulated in WT, TLR2-D and CD14-D animals following CASP, albeit not significantly (Fig. 2D). The up-regulation of the inducible NO-synthase (iNOS; NOS2) can lead to a reduced vascular tone, therefore iNOS mRNA expression following CASP was checked. Interestingly, iNOS was significantly up-regulated only in TLR2-D animals compared to all other groups (Fig. 2E).

### Polymicrobial Sepsis Induced a Profound Loss of Vascular Contractility

To determine vascular contractility during polymicrobial sepsis, we measured the vessel’s response to ascending doses of phenylephrine (PE) 18 h after CASP surgery. Sham-operated WT compared to untreated animals showed a non-significant reduction of vascular contractility of 17.86% (Fig. 3A, F). CASP surgery, in contrast to sham-surgery, induced a significant loss (45.8%) of vascular tone, resulting in decreased maximal response, and decreased hill-slope as well as increased EC50 (Fig. 3A, F). Parameters of all sigmoidal-concentration-response curves to PE are collected in Table S1.

### TLR-deficiency Influences CASP-induced Vascular Dysfunction

Untreated TLR2-D mice showed a significantly reduced vascular contractility following α_1_-stimulation compared to mice from all other strains (Tab. 1; supplement). CASP induced a significant impairment of vascular contractile force with an overall loss of 70.12% in TLR2-D animals compared to their respective controls (Fig. 3B, F). This was the most severe reduction of contractility of all groups. TLR4-D mice showed only a slight albeit significant reduction of contractile force after 18 h following CASP (−26.49% vs. control, Fig. 3C, F).

In contrast, CASP-operated TLR9-D animals did not respond differently to PE compared to their respective controls (–12.83% vs. control, Fig. 3D, F). Our findings provide evidence that TLR9 might be a very important receptor for the induction of vascular dysfunction in polymicrobial sepsis, since TLR9-D mice preserved vascular contractility.

There were significant differences in the vascular contractile response of untreated CD14-D mice compared to the untreated WT, TLR2-D and TLR4-D animals, visible in a higher EC50 (Table S1). Thus, this mouse strain seems to react less sensitive to α_1_-agonist stimulation than all other groups. With respect to polymicrobial sepsis CD14-D mice reacted comparable to TLR9-D animals, i.e. vascular tone did not change significantly following CASP surgery (−13.39% vs. controls, Fig. 3D, E, F). Therefore, signaling through CD14 appears to be similarly crucial to mediate the polymicrobial stimulus as TLR9.

### Vascular Contractility After TLR2 Stimulation

Since in TLR2-D mice vascular tone was already strongly reduced in the control group, we were interested in the effects of specific TLR2 stimulation with LTA in WT mice. Application of LTA lowered the EC50 of phenylephrine leading to a small but significant improvement of contractile function of the vessel wall in the intermediate concentration range of phenylephrine (Fig. 4). Maximal force remained unchanged (not shown).

### TLR9 Antagonism Attenuated Vascular Dysfunction during CASP-induced Sepsis

As TLR9-D mice were protected against CASP-induced injury, we determined whether pharmaceutical TLR9-antagonism might induce similar results in WT animals. Therefore, we injected the synthetic CpG-ODN H154-thioate into the tail vein of WT animals 24 h prior to CASP procedure and measured the vascular contractile properties and aortic cytokine levels 18 h following surgery. Application of H154-thioate without CASP did neither alter the mRNA expression of pro- and anti-inflammatory cytokines nor that of iNOS. Injecting H154-thioate prior to CASP prevented increased expression of all cytokines, thus abolishing the effect of CASP (Fig. 5A–E). Consequently, the loss of vascular contractility during polymicrobial sepsis did not occur (Fig. 5F). Instead we observed a non-significant gain of function, with a 20% increase of contraction force in the H154-thioate pre-treated animals.

## Discussion

The aim of this study was to elucidate the impact of polymicrobial sepsis on vascular contractility and to assess the role of different Toll-like receptors in this setting. It is well known that specific TLR-stimulation increases the expression of pro-inflammatory cytokines like TNF-α, IL-1β and IL-6. This is associated with the development of cardiac dysfunction [21]. However, this study demonstrates for the first time that TLR9 and CD14 play a major role in the development of vascular dysfunction in polymicrobial sepsis.

The CASP surgery applied here resulted in a profound release of bacteria into the peritoneal cavity demonstrated by a significant increase of colony forming units. Thus, a polymicrobial infection in the peritoneum could be induced. This was based on both Gram-positive and Gram-negative bacteria. The bacterial challenge might have caused a release of PAMPs and inflammatory mediators into the vascular system. In WT mice this elicited a local inflammation in the vessel wall (i.e. significant up-regulation of TNF-α) resulting in vascular dysfunction. Hence, the applied model seems to be able to mimic the clinical picture of polymicrobial sepsis. We were unable to observe any significant differences in the amount of gram-positive or gram-negative bacteria in the peritoneal cavities of WT, TLR2-D and TLR9-D following CASP, despite these strains showing the most obvious differences in their susceptibility to polymicrobial sepsis. Hence, it is likely to speculate that differences in the microbiota, resulting from the loss of TLRs, cause the observed effects. However, other groups did not detect significant differences in the microbiota of various TLR knockout strains except for TLR5-D, not used in our study [22], [23].

One of the key players for inducing vasoplegia after endotoxemia is nitric oxide (NO), produced by the inducible NO-synthase (iNOS, NOS2) [13]. However, aortal iNOS mRNA expression was not induced in WT mice after CASP, implying another, iNOS-independent, pathway since these animals also exhibited significantly lower vascular contraction force. Vasorelaxation might in this case rely on activation of constitutively expressed NO-synthases and/or on induction of adrenomedullin, a potent vasodilator, whose expression has been shown to be up-regulated in endotoxemia [8], the release of the vasodilator kynurenine [24] or on COX2-derived thromboxane [25]. Other groups have also demonstrated that in a rat CLP model, contrary to endotoxemia, iNOS protein levels remain unchanged [26]. Furthermore, Vromen and colleagues showed that the level of iNOS activation is responsible for increased vasorelaxation and that iNOS specific inhibition in polymicrobial sepsis had no therapeutic benefit [27]. Hence, the absence of an up-regulation of iNOS following CASP in our WT mice might be a model-dependent issue. TLR2-D animals were the only group showing up-regulation of iNOS following CASP, which might cause their drastic phenotype.

To clarify the role of single PRRs during polymicrobial sepsis, we compared WT mice with those deficient of the investigated PRRs. In this setting, reduced reaction of a PRR-deficient mouse strain to bacterial stimuli means that the respective receptor is of importance in the signaling cascade of the inflammatory stimulus, i. e. in our experiments aortic rings from TLR4-deficient mice showed a 50% loss of contractile function after CASP compared to WT animals. Hence, 50% in loss of contractile function might be attributed to Gram-negative signaling in WT mice. Other bacterial compounds not signaling via TLR4 may be responsible for the observed up-regulation of TLR2 and CD14 in TLR4-D mice after CASP. Increased expression of TLR2 seems to be meaningful as this receptor is important for Gram-positive signaling. CD14 has been described as soluble LPS receptor and TLR4-coreceptor [28]. This view is based on earlier findings by Haziot [29] and Knuefermann [5] showing that CD14-deficiency leads to LPS insensitivity. At the first glimpse the up-regulation of CD14 might be taken as a compensatory mechanism for the missing TLR4. However, CD14 has further functions, which are discussed in detail below.

TLR9 signaling revealed to be decisive during polymicrobial sepsis as vascular tone of TLR9-D mice remained unaffected while that of WT, TLR2-D, and TLR4-D mice was significantly reduced. Furthermore, in mice lacking TLR9 the pro-inflammatory mediators TNF-α, IL-1β and IL-6 were not raised by CASP. The baseline expression of PRRs was not influenced by the gene ablation of TLR9 itself. To test the role of TLR9 in WT mice we injected these with the TLR9-specific antagonist H154-thioate. TLR9-antagonism prevented the loss of vascular contractility and also diminished the expression of pro-inflammatory cytokines significantly. Our results complement the finding of Yasuda and colleagues, that TLR9-antagonism was suitable to reduce sepsis-induced kidney failure [16].

In TLR9-D animals other PRRs are expressed. Therefore, a complete block of the inflammatory signal should not be expected. Interestingly, TLR9-deficiency was able to protect completely against polymicrobial challenge underlining the crucial role of TLR9 in vascular signaling. Since TLR4-D mice express TLR9 they were only partially protected. Thus, the effects of single TLRs are not simply additive during polymicrobial sepsis. Taken together our data give for the first time evidence that TLR9 is a key element in the development of vascular dysfunction during polymicrobial sepsis and might be a pharmacological target. Similar to the TLR-9 gene ablation CD14-deficiency protected vascular contractility completely against polymicrobial challenge. The pro-inflammatory cytokines as well as PRRs were also regulated comparably to TLR9-D animals. Recently, it has been demonstrated, that CD14 acts also as co-receptor for other TLRs than TLR4, including TLR7 and −9 [30]. In our setting, this means that the absence of CD14 interrupts TLR9- as well as TLR4-dependent signaling, thus preventing inflammation-dependent loss of vascular tone. This is consistent with our observation that during CASP CD14 is up-regulated in all investigated TLR-D genotypes (Fig. 1D) and that CD14-deficiency is responsible for complete protection from loss of vascular contractile function (Fig.3E, F).

In contrast to the beneficial effects of CD14- and TLR9-deficiency TLR2-D mice developed an even significantly lower vascular tone than WT animals after CASP (Tab. 1; supplement). Under baseline conditions TLR2-deficiency is accompanied by compensatory up-regulation of all other examined PRRs. Additionally, baseline vascular contractility was reduced in these animals. During CASP, all pro-inflammatory cytokines as well as iNOS were elevated to a level significantly above all other genotypes. This may be due to the elevated PRRs. The overexpression of TLR4, -9 and CD14 in TLR2-D mice might obscure possible effects conveyed by TLR2. Thus, we tested TLR2-specific stimulation with purified LTA. Interestingly, this did not reduce the vascular tone, which supports the view that loss of vascular contractility detected in TLR2-D aortic rings is caused by overexpression of other PRRs. Interestingly, Ha et al. were recently able to show that TLR2 agonists might protect the heart from dysfunction during polymicrobial sepsis [31]. This is in accordance with our findings in the blood vessel.

However, work by Zou and colleagues suggest protective effects of TLR2 knockdown during polymicrobial sepsis. Their data on TLR2-deficient mice undergoing CLP exhibited lower decreases of mean arterial blood pressure, increased survival, limited increases in circulating and cardiac tissue cytokines and maintained left ventricular function compared to wildtype animals [32]. Their findings might be perceived as being contradictory to ours; however, they only observed cardiac function and not vessel function or vessel cytokines. Furthermore, their TLR2 deficient mice also developed lowered mean arterial blood pressure when subjected to CLP. This loss of blood pressure might be due to the observed loss of vascular contractility found here. Their observations on load dependent parameters were derived from Langendorff perfused hearts with constant preload, which is, as Zou et al. point out “not achievable in an *in vivo* septic condition”.

It has been described, that TLR2-deficient mice are more prone to infections, in particular of *Streptococcus pneumoniae* [33] and *Staphylococcus aureus* [34]. Our own findings in combination with those by Ha and colleagues now give evidence that TLR2-deficiency is significant for polymicrobial infections as well, indicating a higher susceptibility for a wide variety of pathogen induced effects.

The experiments of this study demonstrated that TLR4-deficiency protected partly and TLR9- as well as CD14-deficiency protected completely from sepsis-induced vasoplegia. Consequently, the significant up-regulation of TLR4, TLR9 and CD14 in TLR2-D mice may explain their severe reaction to polymicrobial stimuli. Our findings in regard to TLR2 and -4 seem to be in contrast to findings from Williams et al. who found that CLP, but also sham surgery, induces TLR2 and TLR4 expression in a time-dependent manner in the lung, liver and spleen [12]. These differences might be due to the experimental approach (CASP vs. CLP), different mouse strains and the diverse organ systems examined.

The observed higher expression of TLR4, TLR9 and CD14 in TLR2-D mice might be considered a non-physiological knockout phenomenon, but in our understanding it is of clinical relevance since there are several TLR2 polymorphisms in humans, leading to non-functional variants of TLR2 [35]. These patients show a decreased cellular response to lipoproteins and TLR2 gene mutations might also predispose for bacterial infections [36]. In addition, TLR2 ligands might be protective against cardiac [31] and vascular dysfunction in polymicrobial sepsis. Thus, the absence of TLR2 might further aggravate septic cardiac dysfunction.

Another aspect influencing vascular contractility in sepsis is the Ang1/Ang2/Tie-2 receptor axis. Differences in the expression of these factors/receptors have been shown to be important for sepsis induced mortality [37], [38] and to alter blood vessel response to vasoconstrictors [39]. Further studies are needed to better understand the role of these factors in polymicrobial sepsis, specifically looking for alterations of their expression attributable to TLR knockdown.

In summary, our study provides evidence that polymicrobial sepsis leads to vascular dysfunction, possibly independent of iNOS. It also demonstrates that the absence of TLR2 appears to lead to a compensatory increase of other PRRs in the blood vessel concomitant with an increased susceptibility to polymicrobial sepsis with severely impaired vascular tone. We show for the first time, that functional CD14 and TLR9 are crucial for reducing vascular function and mediating the polymicrobial stimulus and that TLR9 antagonism results in improved vascular tone and reduced pro-inflammatory cytokine levels in the vessel wall.

## Supporting Information

Table S1 **Analysis of aortic sigmoidal dose-response curves for Phenylephrine induced contractions.**
(DOC)
