# Supplementary material for: Vascular Dysfunction following Polymicrobial Sepsis: Role of Pattern Recognition Receptors
Source: PLoS One. 2012 Sep 7;7(9):e44531. doi: 10.1371/journal.pone.0044531 (PMC3436884; doi:10.1371/journal.pone.0044531)
Supplement: Table S1 — Analysis of aortic sigmoidal dose-response curves for Phenylephrine induced contractions. (DOC) [file pone.0044531.s001.doc]

**Supplemental information**

**Table 1**

|  | **WT** | | | **TLR2-D** | | **TLR4-D** | |
| --- | --- | --- | --- | --- | --- | --- | --- |
| *Sigmoidal dose-response (variable slope) best-fit values* | *Control* | *Sham* | *CASP* | *Control* | *CASP* | *Control* | *CASP* |
| **BOTTOM** | 9.761 | 10.01 | 9.863 | 10.03 | 10.03 | 10.14 | 10.42 |
| **TOP** | 23.35 | 21.19 | 17.52 | 19.06 | 12.77 | 23.61 | 20.63 |
| **LOGEC50** | -7.316 | -6.989 | -6.848 | -7.149 | -6.645 | -7.301 | -6.72 |
| **HILLSLOPE** | 1.08 | 0.832 | 0.857 | 1.147 | 0.8907 | 1.039 | 1.014 |
| **EC50** | 4.83E-08 | 1.02E-07 | 1.42E-07 | 7.10E-08 | 2.26E-07 | 5.00E-08 | 1.90E-07 |
| **LOGEC50** | 1 | 1 | 1 | 1 | 1 | 1 | 1 |
| **TOP** | 2, 3 | 2, 3 | 2 | 2, 3 |  | 2, 3 | 2, 3 |
| **HILLSLOPE** | n.s all |  |  |  |  |  |  |
|  |  |  |  |  |  |  |  |
|  | **TLR9-D** | | **CD14-D** | | **WT + H154-thioate** | |  |
| *Sigmoidal dose-response (variable slope) best-fit values* | *Control* | *CASP* | *Control* | *CASP* | *Control + H154-thioate* | *CASP + H154-thioate* |  |
| **BOTTOM** | 9.591 | 9.668 | 9.731 | 10.07 | 9.959 | 10.15 |  |
| **TOP** | 23.04 | 21.82 | 22.86 | 22.52 | 20.9 | 23.01 |  |
| **LOGEC50** | -7.013 | -6.773 | -6.727 | -6.096 | -6.865 | -7.092 |  |
| **HILLSLOPE** | 0.9421 | 0.847 | 0.6543 | 0.6632 | 0.9063 | 0.7975 |  |
| **EC50** | 9.70E-08 | 1.69E-07 | 1.87E-07 | 8.01E-07 | 1.36E-07 | 8.08E-08 |  |
| **LOGEC50** | 1 | 1 | 1 |  | 1 | 1 |  |
| **TOP** | 2, 3 | 2, 3 | 2, 3 | 2, 3 | 2, 3 | 2, 3 |  |
| **HILLSLOPE** |  |  |  |  |  |  |  |
|  |  |  |  |  |  |  |  |
|  | significant differences | |  |  |  |  |  |
| 1 | vs CD14-D CASP |  |  |  |  |  |  |
| 2 | vs TLR2-D CASP |  |  |  |  |  |  |
| 3 | vs WT CASP |  |  |  |  |  |  |

**Table 1: Analysis of aortic sigmoidal dose-response curves for Phenylephrine induced contractions**. TOP and BOTTOM form the maximum and minimum of the calculated curves. There was a significantly lower TOP in TLR2-D animals after CASP compared to all other groups and in WT CASP animals compared to all other groups except TLR2-D CASP. LOGEC50 was significantly higher in CD14-D animals after CASP surgery compared to all other groups. (1: p<0.05 vs. TLR2-D CASP; 2: p<0.05 vs. WT CASP; 3: p<0.05 vs. TLR2-D Control; 4: p<0.05 vs. CD14-D CASP; n≥5 animals in each group; mean ± SEM).
